# MRI-Based Radiomics Predicts Tumor Response to Neoadjuvant Chemoradiotherapy in Locally Advanced Rectal Cancer

**DOI:** 10.3389/fonc.2019.00552

**Published:** 2019-06-26

**Authors:** Xiaoping Yi, Qian Pei, Youming Zhang, Hong Zhu, Zhongjie Wang, Chen Chen, Qingling Li, Xueying Long, Fengbo Tan, Zhongyi Zhou, Wenxue Liu, Chenglong Li, Yuan Zhou, Xiangping Song, Yuqiang Li, Weihua Liao, Xuejun Li, Lunquan Sun, Haiping Pei, Chishing Zee, Bihong T. Chen

**Affiliations:** ^1^Department of Radiology, Xiangya Hospital, Central South University, Changsha, China; ^2^Postdoctoral Research Workstation of Pathology and Pathophysiology, Basic Medical Sciences, Xiangya Hospital, Central South University, Changsha, China; ^3^Department of General surgery, Xiangya Hospital, Central South University, Changsha, China; ^4^Department of Radiation Oncology, Xiangya Hospital, Central South University, Changsha, China; ^5^Department of Neurosurgery, Xiangya Hospital, Central South University, Changsha, China; ^6^Department of Radiology, Zhuzhou 331 Hospital, Zhuzhou, China; ^7^Department of Pathology, Xiangya Hospital, Central South University, Changsha, China; ^8^Department of Cardiology, Xiangya Hospital, Central South University, Changsha, China; ^9^Department of Radiology, Keck School of Medicine, University of Southern California, Los Angeles, CA, United States; ^10^Department of Diagnostic Radiology, City of Hope National Medical Center, Duarte, CA, United States

**Keywords:** locally advanced rectal cancer (LARC), neoadjuvant chemoradiotherapy (nCRT), treatment response, magnetic resonance imaging (MRI), machine learning radiomics

## Abstract

**Background:** Conventional methods for predicting treatment response to neoadjuvant chemoradiotherapy (nCRT) in patients with locally advanced rectal cancer (LARC) are limited.

**Methods:** This study retrospectively recruited 134 LARC patients who underwent standard nCRT followed by total mesorectal excision surgery in our institution. Based on pre-operative axial T2-weighted images, machine learning radiomics was performed. A receiver operating characteristic (ROC) curve was performed to test the efficiencies of the predictive model.

**Results:** Among the 134 patients, 32 (23.9%) achieved pathological complete response (pCR), 69 (51.5%) achieved a good response, and 91 (67.9%) achieved down-staging. For prediction of pCR, good-response, and down-staging, the predictive model demonstrated high classification efficiencies, with an AUC value of 0.91 (95% CI: 0.83–0.98), 0.90 (95% CI: 0.83–0.97), and 0.93 (95% CI: 0.87–0.98), respectively.

**Conclusion:** Our machine learning radiomics model showed promise for predicting response to nCRT in patients with LARC. Our predictive model based on the commonly used T2-weighted images on pelvic Magnetic Resonance Imaging (MRI) scans has the potential to be adapted in clinical practice.

**Novelty and Impact Statements:** Methods for predicting the response of the locally advanced rectal cancer (LARC, T3-4, or N+) to neoadjuvant chemoradiotherapy (nCRT) is lacking. In the present study, we developed a new machine learning radiomics method based on T2-weighted images. As a non-invasive tool, this method facilitates prediction performance effectively. It achieves a satisfactory overall diagnostic accuracy for predicting of pCR, good response, and down-staging show an AUC of 0.908, 0.902, and 0.930 in LARC patients, respectively.

## Introduction

Rectal cancer is a common malignancy worldwide, accounting for ~30–50% of colorectal cancer (1, 2). Moreover, in rectal cancer patients, lesions are usually located in middle-low rectum, which causes increased difficulty in treatment and worse prognosis, especially the locally advanced rectal cancer (LARC, T3-4 or N+) (3, 4). Currently, neoadjuvant chemoradiotherapy (nCRT) followed by total mesorectal excision is the recommended treatment for LARC patients, especially those with lesions located in the middle-low rectum (5). The advantages of nCRT are usually significant (6, 7). However, the response of LARC to nCRT varies widely, ranging from pathological complete response (pCR, ypT0N0M0) with no viable tumor cells left in the surgical specimen, to virtually no tumor regression at all (stable) or even tumor progression in a small group of patients (8, 9). Among these patients, pCR is not only associated with favorable disease-free and overall survival (7, 10), but also motivates the “watch-and-wait” treatment strategy, a non-operative option for patients achieving clinical complete response (11). Therefore, clinicians are motivated to identify ways to accurately predict patients' individual responses to nCRT.

Radiological examination has been considered to be one of the means most likely to accomplish this task (12). Among all modalities, Magnetic Resonance Imaging (MRI) is regarded as the most promising method because it uses no radiation, shows high soft tissue resolution, and has wide routine clinical application for evaluation of rectal cancer. Notably, some conventional and functional MRI methods have been reported to show some advantages in predicting tumor response to nCRT (13–15). Unfortunately, conventional MRI analysis remains limited when predict treatment response in individual patient using experience (16). There is a need to develop new methods.

Quantitative image data analysis, such as texture analysis and radiomics are procedures for converting clinical images into high-dimensional, exploitable, and quantitative imaging features by high-throughput extraction of data-characterization algorithms (17). In addition to clinical outcomes, the biomedical information contained in medical images, such as overall information about phenotype and microenvironment of the tumor, may be vitally important for evidence-based clinical decision support. In theory, all magnetic resonance images in different can be used as a source of analysis. In theory, for quantitative analysis, used features can be extracted from images of all modalities (12, 16, 18–22). However, T2 weighted image is almost the most widely used one, when considering the wide availability of images which can be stably acquired based on different machines. Quantitative image data analysis methods have the potential to reveal such biomedical information, providing an opportunity to improve decision-support in oncology and non-invasively (17, 23). The potential advantage of this kind of method has already been verified in colorectal cancer (24) and a variety of other cancers, including nasopharyngeal carcinoma (25), lung cancer (17), and breast cancer (26). Recently, some independent studies (12, 19–22, 24) reported that a multimodality MRI based radiomics model could predict RC tumor response to nCRT with an improved accuracy for pCR and good response prediction. However, due to the relatively small sample size, or the inclusion of multimodality images with other MRI sequences such as diffusion-weighted imaging, or the lack of integration of important relevant clinical pathological features, there is a need for improving accuracy of the prediction model.

In the present study, we retrospectively collected 134 consecutive surgically and pathologically confirmed LARC patients who received standard nCRT before surgery. We developed a machine learning radiomics model based on imaging data extracted from the T2-weighted images, and validated its prediction efficiency of treatment response to nCRT in patients with LARC.

## Materials and Methods

### Patients

This retrospective study was approved by our institutional review board (IRB No. 201610070). The written informed consents from patients were waived.

**Figure 1:**
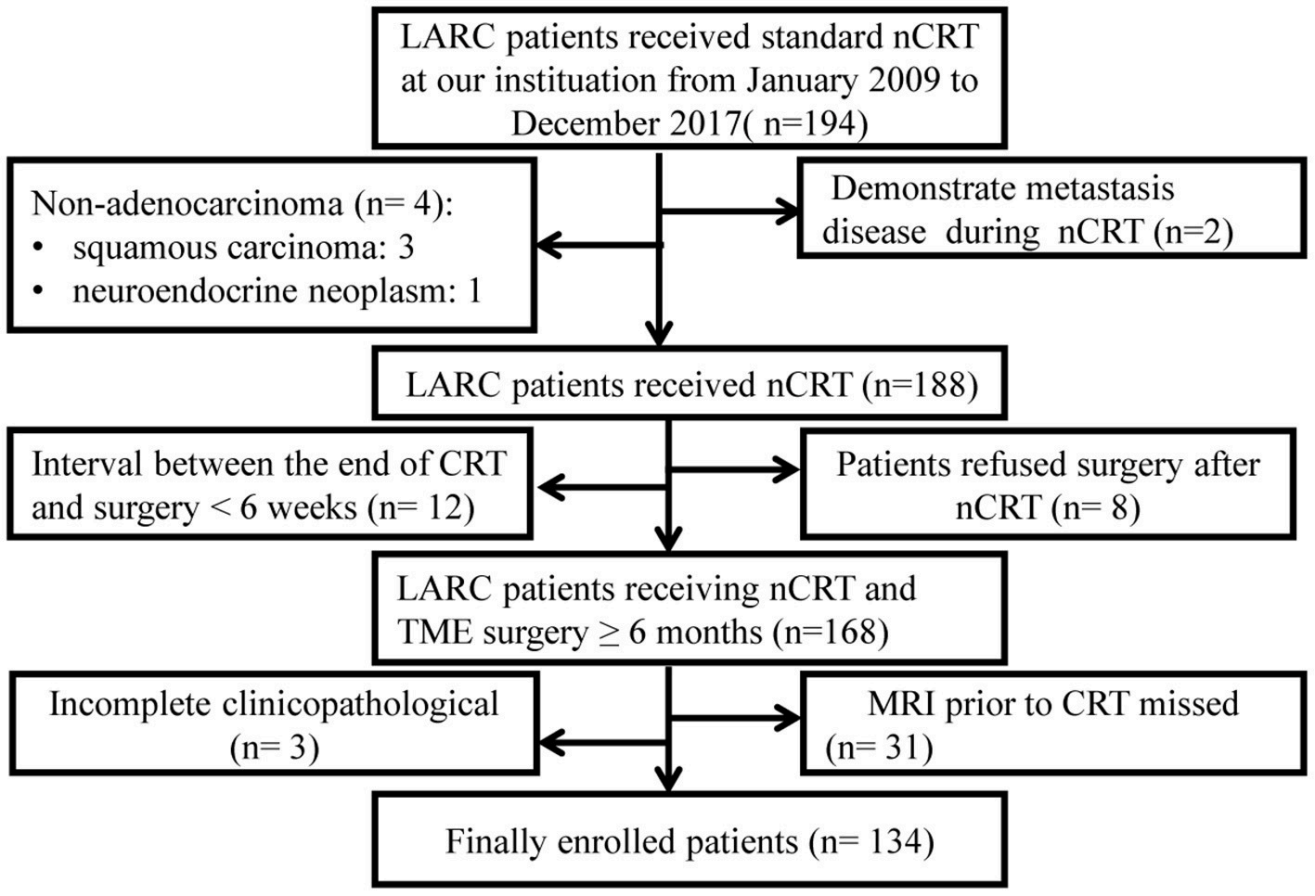
Flow-chart. LARC, Locally advanced rectal cancer; nCRT, neoadjuvant chemoradiotherapy; TME, total mesorectal excision.

Medical data of consecutive biopsy-proven rectal adenocarcinoma patients with LARC treated with nCRT followed by total mesorectal excision between March 2009 and December 2017 in our institution were retrospectively analyzed. Complete clinical data, including MRI imaging of all patient's performed before radiotherapy, was analyzed. Details about the inclusion and exclusion criteria, clinical and pathological characteristics, and treatments information can be found in Supplementary Files. The patients recruiting process was shown in Figure 1.

### Pathological Assessments of Tumor Samples

Each specimen was sampled and evaluated by two experienced dedicated gastrointestinal pathologists. The two pathologists were both blind to the MRI data and clinical data. Criteria for pCRT and non-pCRT were defined as described in previous reports (8). We also classified TRG 3–4 into the good response (GR) group, and TRG 0–2 into the non-GR group according to Dowrak/Rödel's system (27). Changes in TNM staging were recorded by comparing to cTNM before the surgery, and responses were classed as either down-staging or non-down-staging (stability and progression). Details can be found in the Supplementary Files.

### MRI Image Acquisition

All patients underwent an MRI scan in our hospital with either a 1.5 Tesla (Siemens, Erlangen, Germany) or a 3.0 Telsa scanner (GE, Milwaukee, US), using a phased-array body coil, 3–10 days before the start of chemoradiation. To ensure MRI image quality, a quality assurance check was performed biweekly by a hospital radiological physicist and executed bimonthly by the Siemens or GE engineer, as appropriate, according to the maintenance rules for the MRI scanners in our institute. Axial T2-weighted (T2w fast spin echo sequence) images (T2WI) and T1-weighted (T1w spin echo sequence) images (T1WI) were acquired regularly. Subsequently, multiphase T1w images were obtained before and after contrast injection, using a spoiled gradient echo sequence (LAVA/VIBE sequence). Contrast injection and data acquisition were triggered simultaneously. Briefly, a total of four repetitions were acquired, including one before the contrast injection and three after the injection (at 28, 65, and 120 s). For contrast, generally 90–100 ml of the gadolinium-based contrast media dimeglumine gadopentetate (Magnevist; Schering Diagnostics AG, Berlin, Germany) was administrated intravenously at a rate of 2.5 ml/s through a high pressure injector (Optistar LE, Liebel-Flarisheim Company, OH, USA).

Since all patients had at least three kinds of MRI images (T1WI, T2WI, and enhanced T1WI), MRI images from these three serials were included in the present study.

### MRI Image Analysis

All MRI images of each patient were evaluated independently by two experienced abdominal radiologists (reader 1. C.C with 7 years of experience; reader 2, L.X.Y with 15 years of experience), who were totally blinded to all medical information. Final disagreement was resolved in a panel format including two additional radiologists (L.W.H and Y.X.P). The location and boundary of the tumor were confirmed, tumor size, the distance from the lower edge of the tumor to the anal canal, and the MRI-based TNM stage were recorded. The findings were recorded by consensus.

### Texture Analysis Feature Extraction

For each patient, an anonymized representative axial T2WI image in which the lesion had the largest cross-sectional area was selected and retrieved from Picture Archiving and Communication System (PACS, Carestrem, Canada) using Digital Imaging and Communications in Medicine (DICOM) Works software (version 1.3.5). Subsequently, each image was transferred to a personal computer and inputted into the texture analysis software (MaZda Version 4.6, Institute of Electronics, Technical University of Lodz, Poland) (28).

Briefly, the process of texture analysis feature extraction was conducted by 2-steps as follows: (a) selecting and retrieving the suitable MRI images, and then (b) outlining the tumors as the regions of interest (ROI), and extracting quantitative texture analysis features by using the texture analysis software (MaZda Version 4.6, Institute of Electronics, Technical University of Lodz, Poland).

Tumors were outlined as a region of interest (ROI) by performing MaZda on T2WI, while using all other image sequences (especially gadolinium-enhanced images) as references in cases where the margin of the rectal lesion was difficult to define on unenhanced images. Briefly, a ROI was delineated initially by following the tumor outline, with notation that fat and air outside the mass are not included. Then, the ROI was saved for subsequent texture analysis. Contouring was performed carefully to cover the maximum extent of the tumor without exceeding the lesion border, to avoid contamination from adjacent normal rectal tissues or the intestinal lumen. For each ROI, a total of 340 quantitative features were automatically generated using MaZda software, including a gray level histogram, gradient, run-length matrix, co-occurrence matrix, autoregressive model, and wavelet transform analysis according to the software settings.

### Evaluation of the Reproducibility of Radiomics Feature Extraction by the Two Radiologists

The reproducibility assessment of the features extracted by the two radiologists from the independent segmentations of T2WI images of all patients was performed. The inter-observer (reader 1 v reader 2) and intra-observer (reader 1 twice) correlation coefficient values were evaluated. The final consistency is evaluated by the following criteria regarding the correlation coefficient values: <0.20 indicates poor reproducibility, 0.21–0.40 fair reproducibility, 0.40–0.60 moderate reproducibility, 0.61–0.80 good reproducibility, and 0.81–1.00 excellent reproducibility. Generally, a correlation coefficient >0.75 is regarded as being in good agreement.

For the Kappa consistency test, excellent, good, and poor agreement were defined as kappa values of >0.81, in the range of 0.61–0.80, and <0.60, respectively.

The Mann-Whitney *U*-test was used to compare the values of each feature between the two groups. An independent samples *t*-test or Kruskal-Wallis *H* test, where appropriate, was used to assess the differences between the features generated by reader 1 (first time) and those generated by reader 2, as well as between the features generated twice by reader 1.

Inter-observer and intra-observer reproducibility of texture feature extraction was initially analyzed with 50 randomly chosen images from all T2WI images selected for evaluation by the two radiologists (reader 1, and reader 2). To assess the intra-observer reproducibility, reader 1 repeated the generation of texture features twice within a 2-week period following the same procedure. Reader 1 completed the workflow for the remaining images.

### Statistical Analysis, Features Selection, Signature Generation, and Prediction Model Building

All statistical analyses were conducted using IBM SPSS version 20.0.0 (IBM Corporation, Armonk, NY, USA). To test the difference between groups, the Wilcoxon rank-sum test was performed for the quantitative features, and the chi-square test or fisher's exact test was performed for the qualitative features.

All data processing, data reduction and feature selection, and model built were performed using MATLAB 2017a (The Mathworks, Inc., Natick, MA, USA). The least absolute shrinkage and selection operator (LASSO) method, was used to select the most useful predictive features from the primary data set, and a radiomics score (Rad-score) was calculated for each patient at the mean time as a linear combination of selected features that were weighted by their respective coefficients. Based on these selected features, another classification model was also constructed by Random forest (RF), and the RF-score was generated. Subsequently, a combined classification model was finally built by the support vector machine (SVM) method (SVM-score), based on Rad-score and RF-score in the previous step. On the basis of two SVM-scores obtained, calculated from TE and TRC features, respectively, a final classification model was generated by using the SVM method again (SVM-score-final). Through the above steps, a total of seven models were generated representing each classification task, considering there are two kinds of data (Texture analysis [TA] features, and Traditional radiological-clinicopathological [TRC] features) that were used to build the model. The seven models include three models generated from TA features (model based on Rad-score, RF model, and first-step SVM model), three models generated from TRC features (model based on Rad-score, RF model, and first-step SVM model), and one combined SVM model.

The basic idea of this algorithm is to consider LASSO and RF as weak regressors and combine them using SVM. For each type of data, i.e., texture feature, we first use LASSO to obtain the Rad-score, and use its side product, i.e., important features, as the feature set of RF to obtain the RF-score. Since Rad-score and RF-score are independently acquired by two different weak regressors, using SVM to regress them in a two- dimensional plane achieves a better result than by them owns. Moreover, data sets Texture feature and Traditional radiological-clinicopathological data are also independent to each other. So for the same reason, we use SVM to regress the scores from Texture feature and Traditional radiological-clinicopathological data to get the final regression score. Finally, the regressed scores can be binarized for further prediction.

**Figure 2:**
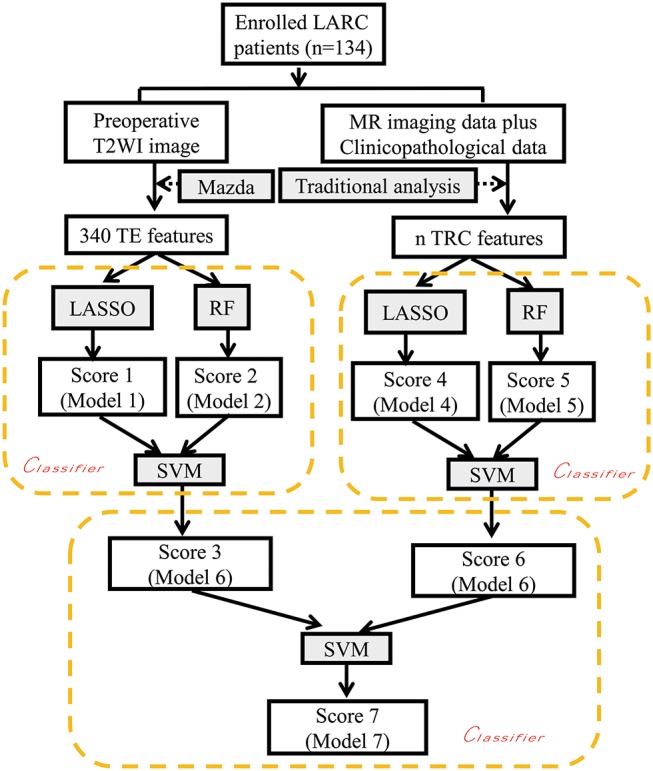
Flow chart depicting construction of the classification models. LARC, locally advanced rectal cancer; TE, texture features; TRC Traditional radiological features and clinicopathological features; LASSO, least absolute shrinkage and selection operator; RF Random forest; SVM support vector machine. The *n*_x_ or *n*_y_ terms used here indicate the different numbers of selected features used in the LASSO method with three different reduction schemes based on 340 TE features and 31 TRC features, respectively. The texture analysis software used was MaZda Version 4.6 (Institute of Electronics, Technical University of Lodz, Poland).

To evaluate the performance of the models, all patients were divided into two cohorts: a training cohort and a validation cohort. The models were developed in the training cohort, and tested in the validation cohort. The classification efficiencies of each kind of model mentioned above, including the receiver operating characteristic (ROC) curves, both in the training and validation cohort were calculated. A *P*-value < 0.05 was considered statistically significant. Details of the flow chart for building the classification model are shown in Figure 2.

## Results

### Patients Characteristics

There were134 patients enrolled in the present study. Patients' characteristics in the training and validation cohorts were summarized in Table 1. Patients were randomly allocated into training cohort and validation cohort in a 3:1 ratio for building the pCR predictive model, the down-staging model, and the good response model. No differences were found between the training and validation cohorts in any of the three models. In addition, the patients' clinicopathological data mostly consisting of laboratory data were also used in building the predictive models.

**Table 1:** Clinicopathological characteristics in three tumor response predictive models.

	**Down-staging predictive model**			**pCR predictive model**			**Good response predictive model**		
	**Training cohort**	**Validation cohort**	***P*-value**	**Training cohort**	**Validation cohort**	***P*-value**	**Training cohort**	**Validation cohort**	***P*-value**
**Gender**									
Male	54 (57.4%)	26 (65.0%)	0.415	57 (61.3%)	23 (56.1%)	0.572	55 (58.5%)	25 (62.5%)	0.667
Female	40 (42.6%)	14 (35.0%)		36 (38.7%)	18 (43.9%)		39 (41.5%)	15 (37.5%)	
Age (years)	50.62 ± 10.29	54.70 ± 10.74	0.040	52.32 ± 10.68	50.73 ± 10.32	0.424	52.02 ± 10.82	51.4 ± 10.04	0.757
Distance from the anal verge (cm)	5.00 (3.00–6.00)	5.30 ± 2.15	0.264	5.0 (4.0–6.0)	5.00 (3.00–6.50)	0.185	5.00 (3.38–6.25)	5.00 (3.00–6.00)	0.233
**Pathology type**			0.463			0.320			0.087
Well/moderately differentiated adenocarcinoma	70 (74.5%)	33 (82.5%)		71 (76.3%)	32 (78.0%)		70 (74.5%)	33 (82.5%)	0.087
Poor differentiated adenocarcinoma	17 (18.1%)	6 (15.0%)		18 (19.4%)	5 (12.2%)		20 (21.3%)	3 (7.5%)	
Mucinous carcinomas	7 (7.4%)	1 (2.5%)		4 (4.3%)	4 (9.8%)		4 (4.3%)	4 (10.0%)	
**Clinical T staging (cT)**			1.000			1.000			0.364
cT2	3 (3.2%)	0		2 (2.2%)	1 (2.4%)		1 (1.1%)	2 (5.0%)	
Ct3	73 (77.7%)	29 (72.5%)		71 (76.3%)	31 (75.6%)		72 (76.6%)	30 (75.0%)	
cT4	18 (19.1%)	11 (27.5%)		20 (21.5%)	9 (22.0%)		21 (22.3%)	8 (20.0%)	
**Clinical N staging (cN)**			0.632			0.847			0.540
cN0	18 (19.1%)	10 (25.0%)		17 (18.3%)	11 (26.8%)		19 (20.2%)	9 (22.5%)	
cN1a	18 (19.1%)	10 (25.0%)		20 (21.5%)	8 (19.5%)		18 (19.1%)	10 (25.0%)	
cN1b	25 (26.6%)	8 (20.0%)		23 (24.7%)	10 (24.4%)		26 (27.7%)	7 (17.5%)	
cN1c	1 (1.1%)	0		1 (1.1%)	0		1 (1.1%)	0	
cN2a	20 (21.3%)	5 (12.5%)		19 (20.4%)	6 (14.6%)		15 (16.0%)	10 (25.0%)	
cN2b	12 (12.8%)	7 (17.5%)		13 (14.0%)	6 (14.6%)		15 (16.0%)	4 (10.0%)	

### The Classification Model Building and Predicting Efficiency

From a total of 340 features that were extracted from T2-wighted images for each patient, a set of features with corresponding numbers were selected by LASSO and used to calculate the Rad-scores for the pCR, Good Response, and Down-staging models.

#### Predicting Pathological Complete Response (pCR)

**Figure 3:**
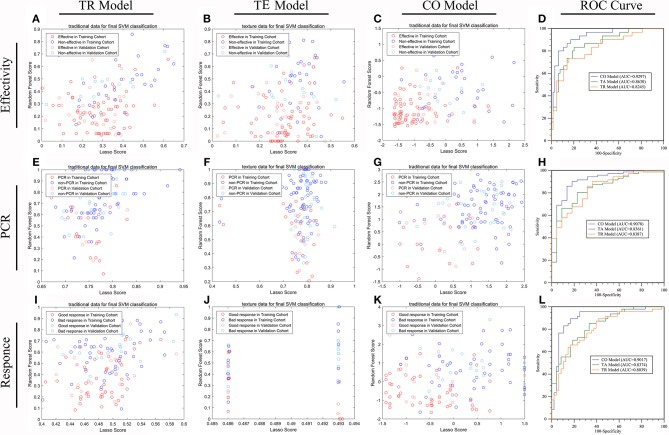
The efficiencies of machine learning models predicting treatment response in LARC patients receiving nCRT. The distribution of patients with down-staging disease or not **(A–D)**, pCR or not **(E–H)**, and good response or not **(I–L)**, in TRC based model (model 6) **(A,E,I)**, TE based model (model 3) **(B,F,J)** and the combined TRC and TE based model (Model 7) **(C,G,K)** were demonstrated by scatter plots. The ROC test **(D,H,L)** shows that the efficiency of model 7 was significantly higher than that of either model 6 or model 3 in all three missions (all *P* < 0.05). There is no significant difference in prediction efficiency between model 6 and model 3 in any of the three missions (all *P* > 0.05).

On the basis of the selected 10 texture and 8 clinicopathological features, a predictive model was finally constructed with SVM method for pCR prediction. The SVM model yielded an AUC of 90.78% in the training cohort, and 87.45% in the validation cohort (Figure 3 and Supplementary Figure 1).

#### Predicting Good Response (GR)

The predictive model built based on the 10 texture features and 7 clinicopathological features achieved an AUC of 90.17% in the training cohort, and 89.72% in the validation cohort (Figure 3 and Supplementary Figure 1).

#### Predicting Down-Staging

**Figure 4:**
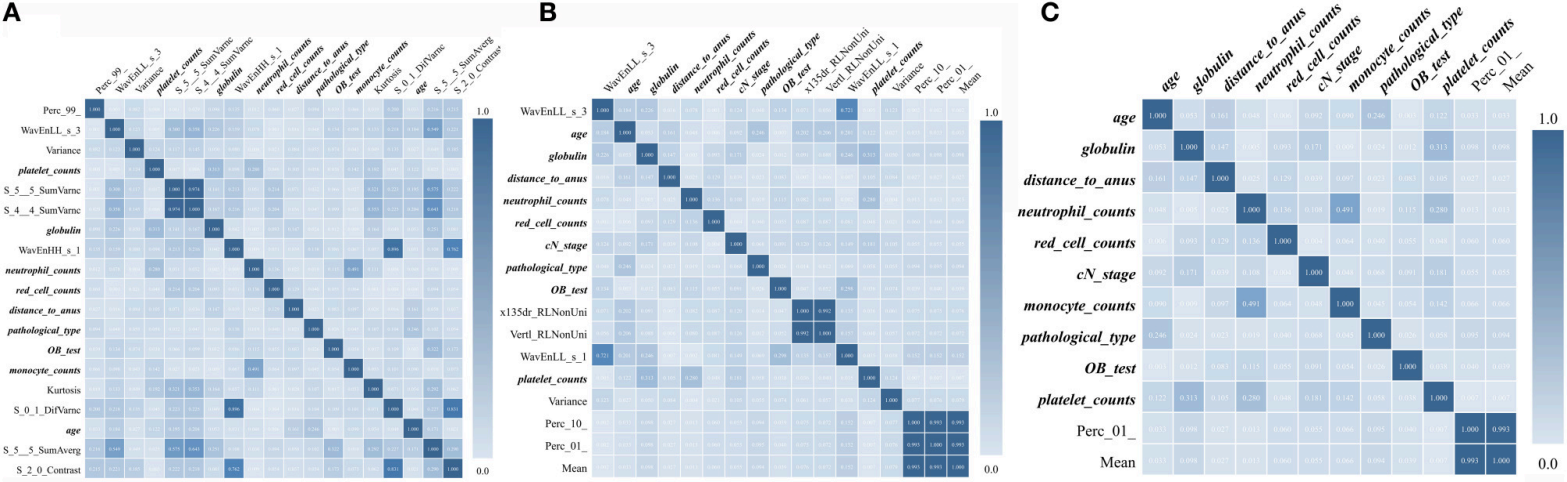
Correlation matrix maps show the correlation among all TE and TRC features used in predictive models. **(A)** Down-staging model. **(B)** PCR model. **(C)** Good-response model. TRC features are expressed in bold fonts.

The predictive model with 10 texture features and 7 clinicopathological features showed an AUC of 92.97% in the training cohort, and 89.20% in the validation cohort (Figure 3 and Supplementary Figure 1). Details about prediction efficiency of three kinds of models could be found in Table 2. The correlation matrix of the selected features used in the three kinds of models was showed as Figure 4.

**Table 2:** The efficience of models to predict the treatment response in LARC petients.

	**Training cohort**			**Validation cohort**		
	**Score 3**	**Score 6**	**Score 7**	**SVM1**	**SVM2**	**SVM3**
**DOWN-STAGING**						
AUC	0.8630 (95% CI: 78.36–94.25%)	0.8245 (95% CI: 73.07–91.83%)	0.9297 (95% CI: 87.62–98.31%)	0.8006 (95% CI: 65.28–94.84%)	0.8462 (95% CI: 72.46–96.77%)	0.8920 (95% CI: 79.40–99.01%)
Specificity	0.82812 (95% CI: 68.750–95.312%)	0.85938 (95% CI: 59.375–96.875%)	0.90625 (95% CI: 73.438–98.438%)	0.7778 (95% CI: 51.85–96.30%)	0.7778 (95% CI: 51.85–96.30%)	0.7778 (95% CI: 62.96–92.59%)
Sensitivity	0.83333 (95% CI: 63.333–96.667%)	0.73333 (95% CI: 56.667–93.333%)	0.9000 (95% CI: 73.333–100.000%)	0.8462 (95% CI: 61.54–100.00%)	0.9231 (95% CI: 69.23–100.00%)	0.9231 (95% CI: 76.92–100.00%)
Accuracy	0.82979 (95% CI: 74.468–89.362%)	0.81915 (95% CI: 69.149–89.362%)	0.89362 (95% CI: 79.787–95.745%)	0.8000 (95% CI: 65.00–92.50%)	0.8250 (95% CI: 67.50–92.56%)	0.8500 (95% CI: 70.00–92.50%)
**PCR**						
AUC	0.8361 (95% CI: 74.13–93.09%)	0.8387 (95% CI: 74.83–92.91%)	0.9078 (95% CI: 83.15–98.41%)	0.8194 (95% CI: 69.08–94.79%)	0.7581 (95% CI: 58.56–93.05%)	0.8745 (95% CI: 74.82–99.49%)
Specificity	0.86364 (95% CI: 63.64–100.00%)	0.77273 (95% CI: 54.55–100.00%)	0.86364 (95% CI: 72.73–100.00%)	1.00 (95% CI: 80.00–100.00%)	0.9000 (95% CI: 40.00–100.00%)	0.9000 (95% CI: 50.00–100.00%)
Sensitivity	0.77465 (95% CI: 54.93–91.55%)	0.85915 (95% CI: 49.30–95.78%)	0.88732 (95% CI: 69.01–97.18%)	0.67742 (95% CI: 48.39–90.32%)	0.67742 (95% CI: 32.26–100.00%)	0.80645 (95% CI: 51.61–100.00%)
Accuracy	0.78495 (95% CI: 64.52–89.25%)	0.82796 (95% CI: 61.29–90.32%)	0.88172 (95% CI: 75.27–94.62%)	0.7561 (95% CI: 60.98–90.24%)	0.73171 (95% CI: 48.78–92.68%)	0.85366 (95% CI: 63.35–95.18%)
**GOOD-RESPONSE**						
AUC	0.8374 (95% CI: 75.95–91.53%)	0.8039 (95% CI: 71.42–89.36%)	0.9017 (95% CI: 83.30–97.05%)	0.7920 (95% CI: 65.24–93.16%)	0.7744 (95% CI: 62.65–92.23%)	0.8972 (95% CI: 80.19–99.25%)
Specificity	0.77083 (95% CI: 54.17–97.92%)	0.8125 (95% CI: 52.08–93.75%)	0.875 (95% CI: 70.83–97.92%)	0.7143 (95% CI: 38.10–100.00%)	0.7143 (95% CI: 38.10–95.24%)	0.8571 (95% CI: 66.67–100.00%)
Sensitivity	0.80435 (95% CI: 50.00–95.65%)	0.73913 (95% CI: 56.52–95.65%)	0.9130 (95% CI: 76.09–100.00%)	0.8947 (95% CI: 47.37–100.00%)	0.8421 (95% CI: 52.63–100.00%)	0.8947 (95% CI: 68.42–100.00%)
Accuracy	0.7766 (95% CI: 70.21–85.11%)	0.7766 (95% CI: 69.15–85.11%)	0.88298 (95% CI: 81.92–93.62%)	0.7500 (95% CI: 65.00–87.50%)	0.7750 (95% CI: 65.00–87.50%)	0.8750 (95% CI: 75.00–95.00%)

## Discussion

To the best of our knowledge, this was the first cohort studied to date utilizing monosequence-MRI-based machine learning radiomics to predict tumor response to neoadjuvant chemoradiation therapy in patients with locally advanced rectal cancer. Our predictive model constructed with both radiomics features and clinicopathological data achieved higher accuracies than previously reported in the literature, with an AUC of more than 90%. Substantial evidence from prior studies has demonstrated that a number of clinicopathologcial and radiological features may help to predict treatment response (16, 18, 29). Nevertheless, no single factor has stood out to be the most reliable way for clinicians to use in decision making process (6, 16). It is important to distinguish the LARC patients who will likely to respond to nCRT from patients who would not. However, this has not been achieved yet. We introduced here a new imaging oriented strategy for a better prediction, which may have potential for clinical practice.

Our study is in general accord with prior research (19, 30, 31). Nie et al. (12) have reported a relatively satisfactory result by using a radiomics method, with an AUC of 0.84 for pCR and 0.89 for good response prediction. Most recently, Cui et al. (19) reported a further attempt on a bigger group LARC patients by similar methods, which show very high predictive efficiency with an AUC of 0.944. In addition, several LARC studies (20, 21) also perform similar radiomics-based studies with good experimental results, using features extracted from multimodality MR images including T2WI. However, there were obvious advantages in our study when compared to these studies. First, we fully evaluated three aspects of the treatment response: not only pCR and good response, but also down-staging. Our study has the potential to provide more information on the tumor and treatment response. Second, the number of enrolled patients in our study (*n* = 134), was larger than that in the Nie's (*n* = 48), and comparable to Cui y's (*n* = 186), which can ensure the desired prediction results. Third, we also included conventional MRI findings and clinicopathological data which may further improve the prediction. Lastly, our radiomic features were extracted from only one sequence, i.e., the T2-weighted images, other than the multi-sequence MRI images used in previous studies. The T2 weighted images are commonly used in clinical practice, which is familiar to radiologists. In addition, it can be acquired easily and the images are quite stable in appearance, especially when compared with images obtained by special sequence, such as diffusion weighted images. Notably, diffusion weighted images are prone to distortion and susceptibility artifacts, which affect tumor segmentation and data extraction. Similarly, other sequences such as T1-weighted dynamic contrast enhanced images depend on the amount and distribution of the injected contrast-enhancing agent, which might be influenced by variable hemodynamic conditions in the patients.

The exact reason for why quantitative MRI-based texture data appear to be able to predict treatment response is still largely unknown. In theory, the biological phenotype of tumors, including treatment response, is largely determined by their underlying molecular subtypes, whose manifestations may vary. One of the phenotypes may be radiological heterogeneity, including inter- and intra-tumor heterogeneity. A large body of literature indicates that texture based radiomic modeling can evaluate tumor heterogeneity, and can correlate radiological findings with underlying genomic and biological characteristics, including prognosis and treatment response (17, 23). Our study may add into the literature in this regard as we have shown a predictive model for treatment response with high accuracy. From another perspective, the large amount of previous evidence (13, 15), supporting using advanced MRI-based radiomic features to predict different responses to nCRT in patients with rectal cancer.

In addition, we introduced clinicopathological features into the prediction model, which may contribute significantly to the improvement of prediction efficiencies. These features may represent, to some extent, some of the intrinsic properties of the tumor (32, 33). For example, the fecal occult blood test and red cell counts may indicate oxygen status of tumor. Neutrophil counts, Monocyte counts, globulin, or platelet counts, may actually reflect the immune status of LARC patients to some extent. The hypoxia and immune status of the tumor can influence tumor treatment response and mediate radiotherapy resistance (34, 35). Pathology type and distance from the anal verge also influence the tumor response, as has been shown in previous studies (36, 37). Our study results suggest that these clinicopathological data may play an important role in treatment response.

There were several limitations in our study. First, as a retrospective study, there may be a selection bias. Second, the sample size in our study was modest, which may affect the accuracy and stability of the predictive models. Third, both the building and validation of the models were conducted in our institution with a single dataset. A multicenter prospective study might be helpful to further validate and optimize our prediction models. The texture features were extracted from the largest cross-sectional area of the tumor rather than from the entire tumor, which may raise questions as to whether these features were optimally representative of the characteristics of the entire tumor. Lastly, the MRI images used in the texture feature extraction were obtained from three different MRI scanners (Siemens and GE) in our hospital, and differences among the scanners may potentially influence the texture features and the subsequent model building. Future research is needed to standardize the signal intensity among different MRI scanners.

## Conclusion

Our study showed a predictive model built with radiomic features and clinicopathological data was promising to predict tumor response to neoadjuvant chemoradiation in patients with locally advanced rectal cancer. In addition, our method developed with information from the clinically obtained T2-weighted sequence may be used as a complimentary tool to assist clinical decision making. Nevertheless, future prospective multicenter studies with larger samples will be needed to validate our study result and to optimize the prediction models for clinical practice.

## Author Contributions

XY, QP, HP, and WeiL conceived and designed the experiments. XY, QP, and BC wrote the paper. All authors analyzed the data and contributed to reagents, materials, analysis tools, and also read and approved the final manuscript.

### Conflict of Interest Statement

The authors declare that the research was conducted in the absence of any commercial or financial relationships that could be construed as a potential conflict of interest.

## Abbreviations

LARC: locally advanced rectal cancer
nCRT: neoadjuvant chemoradiotherapy
MRI: magnetic resonance imaging
RC: rectal cancer
CRC: colorectal cancer
pCR: pathological complete response
cCR: clinical complete response
AUC: area under curve
DWI: diffusion-weighted imaging
GR: good response
ROI: region of interest
ROC: receiver operating characteristic.
