# Supplementary material for: MRI-Based Radiomics Predicts Tumor Response to Neoadjuvant Chemoradiotherapy in Locally Advanced Rectal Cancer
Source: Front Oncol. 2019 Jun 26;9:552. doi: 10.3389/fonc.2019.00552 (PMC6606732; doi:10.3389/fonc.2019.00552)

**Supplement files**

**Patients**

Patients were selected with tumors that were clinical stage T3-4 or N+ (c-Stage Ⅱ-Ⅲ), as evaluated by digital rectal examine, chest X-ray, abdomen, pelvis and/or chest contrast-enhanced computed tomography (CT), pelvic magnetic resonance imaging (MRI), bone single photon emission computed tomography (SPECT), and/or endorectal ultrasonography (ERUS), without any evidence of distant metastases. Exclusion criteria included synchronous tumors, short-course radiotherapy, previous pelvic radiotherapy, and an interval between radiotherapy and surgery shorter than six months.

**Clinical and pathological characteristics**

The clinical characteristics we examined generally included distance of the tumor from the anal verge, blood cell tests, fecal occult blood test (FOBT), liver and kidney function, blood glucose and lipid levels, and serum tumor markers. Specially, the included features were: gender, age, distance of tumor from the anal verge, red cell count, hemoglobin, platelet count, neutrophil count, lymphocyte count, monocyte count, neutrophil to lymphocyte ratio (NLR), lymphocyte to monocyte ratio (LMR), platelet-to-lymphocyte ratio (PLR), red blood cell volume distribution width (RDW), mean platelet volume (MPV), FOBT, albumin, globulin, albumin/globulin (A/G), blood glucose, triglycerides, cholesterol, high-density lipoprotein (HDL), low density lipoprotein (LDL), urea, creatinine, serum Carcinoembryonic antigen (CEA) level, serum CA199 level, and serum CA125 level. Except for FOBT, all the markers were obtained by measuring peripheral blood at diagnosis in the fasting condition. The pathology type was determined from histological biopsy.

**Treatments**

Neoadjuvant radiotherapy was delivered to the whole pelvis at a dose of 46-50Gy in 23-25 fractions (2Gy/fraction, 5 days a week). All patients underwent CT simulation for three-dimensional conformal planning and Intensity Modulated radiotherapy (IMRT), and a three-field treatment plan was used involving a 6-MV photon posterior-anterior field and 15-MV photon opposed lateral fields. Concurrently, capecitabine was taken orally at a dose of 825 mg/m^2^ twice daily during radiotherapy with weekend breaks. During the interval between nCRT and surgery, additional chemotherapy was administrated, the regimens mainly being CapeOX or FOLFOX6. At least six weeks after the completion of radiotherapy, radical surgery was performed, with the surgery type left to surgeon’s discretion, including low anterior resection (LAR), abdominoperineal resection (APR), Hartmann’s operation, and LAR plus prophylactic ileostomy and trans.

**Pathological Assessments of Tumor Response**

After resection, each specimen was sampled, embedded in paraffin, and sliced into 4-μm-thick sections to be evaluated by an experienced pathologist and further reviewed by a dedicated gastrointestinal pathologist, both blinded to the MRI data. A pCR was defined as no viable tumor cells present in the bowel wall (T stage) or regional nodes (N stage)--ypT0N0, equivalent to the tumor regression grade (TRG) 4 based on Dowrak/Röde’s definition: fibrotic mass, hyaline degeneration or acellular mucin pools only, without detectable tumor cells (complete regression). All the other pathological conditions, including TRG 0-3 (no regression, minimal regression, moderate regression and near-complete regression), were defined as non-pCR. We also classified TRG 3-4 into a good response (GR) group, and TRG 0-2 into a non-GR group.

Alteration in TNM staging was also included in the assessment of tumor response. By comparing cTNM before and pTNM after chemoradiotherapy, staging alteration was classed into either down-staging or non-down-staging (stability and progression).

**TNM system**

TNM staging system is the most common tumor staging system in colorectal cancer. T means Tumor, representing the tumor invasion depth of the bowel wall. N means Node, representing the regional lymph node metastasis. M means metastasis, representing distant organ metastasis. In our research, we adopted the 8^th^ edition of the American Joint Committee on Cancer (AJCC) Cancer Staging Manual. T0 means no evidence of tumor in colorectum. N0 means no evidence of metastasis in regional lymph nodes. M0 means no evidence of metastasis in distant organs.

The latest version (the eighth version) of the American Joint Committee (AJCC) on Cancer manual as the colorectal cancer staging system. The details are shown as follows.

T0: No evidence of primary tumor

T1: Tumor invading submucosa

T2: Tumor invading the muscularis propria

T3: Tumor penetrating the muscularis propria and arriving at colorectal fat tissue

T4: Tumor directly invading other organs or structures

N0: No lymph node metastasis and no tumor deposits (TD)

N1: 1-3 lymph nodes metastases

N1a: 1 lymph node metastases

N1b: 2-3 lymph nodes metastases

N1c: Although there was no regional lymph node metastasis, TDs were submucosal, mesangial or peritoneum-covered para-colorectal tissue.

N2: More than or equal to 4 lymph node metastases

N2a: 4-6 regional lymph node metastases

N2b: More than or equal to 7 lymph node metastases

**Equations**

**For Down-staging prediction model**

$$\begin{matrix} S\text{cor}e4=0.0034501\times OB\_Test+(-0.010762)\times age+0.0176\times dis\tan ce\_to\_anus+ \\ 0.002761\times patho\log ical\_type+(-0.005293)\times red\_cell\_counts+(-0.00094983) \\ \times platelet\_counts+(-0.004373)\times neutrophil\_counts+0.00041395\times monocyte\_counts \\ +0.0010542\times globulin \end{matrix}$$

**For PCR prediction model**

$$\begin{matrix} S\text{co}re1(Rad-score)=2.5465\times10^{-7}\times Mean+(-5.581\times10^{-6})\times Variance+ \\ 3.0901\times10^{-8}\times Perc\_01+9.1938\times10^{-8}\times Perc\_10+(-2.3538\times10^{-6})\times\\ Vertl\_RLNonUni+(-4.473\times10^{-6})\times x135dr\_RLNonUni+3.5794\times10^{-5}\times\\ WavEnLL\_s\_1+(-1.1764\times10^{-5})WavEnLL\_s\_3 \end{matrix}$$

**For Good response prediction model**

$\begin{matrix} Score4=0.00096401\times OB\_Test+(-0.0028053)\times cN\_stage+(-0.0030777)\times age+ \\ 0.0040173\times dis\tan ce\_to\_anus+(-0.00016279)\times patho\log ical\_type+ \\ (-0.00019609)\times red\_cell\_counts+(0.00031664)\times platelet\_counts \\ +(-0.00051429)\times neutrophil\_counts+(-0.00074274)\times monocyte\_counts+ \\ 0.00090138\times globulin \end{matrix}$

**Legends**.

Supplementary Figure 1: LARC patients in the training cohort and the validation cohort sorted based on the SVM model constructed by using combined TE and TRC features. A B: Predictive model for down-staging patients. C D: Predictive model for pCR patients. E F: Predictive model for good response patients.


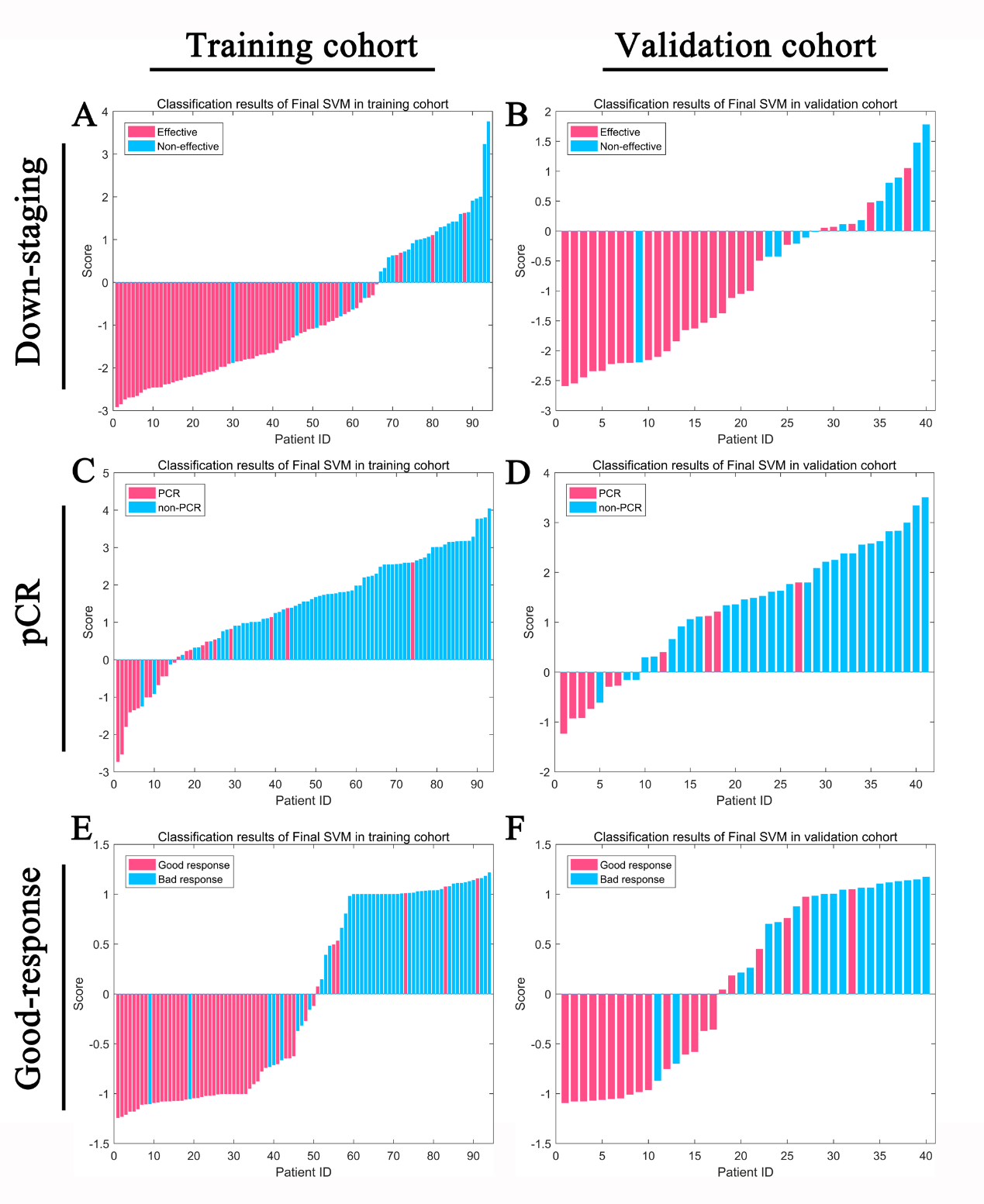

Supplement: Supplementary file 1 [file Data_Sheet_1.docx]
